# Antihypertensive drug treatment in white-coat hypertension: data from the Plaque HYpertension Lipid-Lowering Italian Study

**DOI:** 10.1097/HJH.0000000000003176

**Published:** 2022-07-25

**Authors:** Giuseppe Mancia, Rita Facchetti, Fosca Quarti-Trevano, Guido Grassi

**Affiliations:** a Clinica Medica, Department of Medicine and Surgery; b University of Milano-Bicocca, Milan, Italy

**Keywords:** ambulatory blood pressure monitoring, antihypertensive agents, hypertension, white-coat hypertension

## Abstract

**Aim:**

Little evidence is available on whether antihypertensive treatment lowers cardiovascular risk in white-coat hypertension (WCH). Protection might be indirectly inferred, however, from the blood pressure (BP) effects of treatment as in trials BP reduction is linearly related to outcome reduction. We analyzed the effect of antihypertensive treatment on office and ambulatory BP in WCH using data from the Plaque HYpertension Lipid-Lowering Italian Study (PHYLLIS).

**Methods:**

: Office and ambulatory blood pressure were measured in 470 hypertensive patients randomized to fosinopril or hydrochlorothiazide alone or combined with a statin before treatment and at 6 month or yearly intervals during 2.6 years of follow-up. Patients were divided into two groups according to whether before randomization to treatment office and 24-h mean BP were elevated (sustained hypertension) or office BP was elevated but 24-h BP values were normal (WCH).

**Results:**

: In both sustained hypertension and WCH antihypertensive treatment was associated with an early marked office BP reduction, which persisted virtually unchanged throughout the treatment period. In contrast, 24-h (and day and night) BP showed a marked and persistent treatment-related fall in sustained hypertension but no change in WCH. The results were similar when data were separately analyzed in patients under fosinopril or diuretic, with or without statin treatment.

**Conclusion:**

: In WCH, antihypertensive treatment can effectively and durably reduce office BP. This reduction is accompanied by the inability to lower ambulatory BP from the normal values characterizing this condition at baseline. This appears to be unrelated to the type of treatment employed.

## INTRODUCTION

Following early negative studies [1,2], recent studies and large meta-analyses have shown that white-coat hypertension (WCH), that is, a condition characterized by an elevation of office but not of out-of-office blood pressure (BP), is associated with a risk of cardiovascular outcomes that is less than that of an office and out-of-office BP elevation (sustained hypertension) but significantly greater than that of normotension, that is, office and out-of-office BP normality [3–5]. This has found additional support from the evidence that WCH is accompanied by a metabolic profile and a prevalence of subclinical organ damage that is also intermediate between normotension and sustained hypertension as well as by a risk of developing high cardiovascular risk conditions, such as sustained hypertension and diabetes that is substantially greater than that of individuals with a normal BP [6]. Information is limited, on the other hand, on the effect of antihypertensive treatment on the WCH-associated cardiovascular risk. The post hoc analysis of a subgroup of patients from the Systolic Hypertension in Europe (SYSTEUR) trial has shown that cardiovascular outcomes were not significantly different between WCH patients who were given antihypertensive drugs or placebo, in the context, however, of a low number of events and thus of a statistical power inadequate to reach conclusions [7].

Treatment-induced BP reductions are linearly related with a reduction of cardiovascular outcomes over a wide range of BP values [8,9], which implies that the above limitation might be in part compensated by precise information on the effect of treatment on office and out-of-office BP in WCH individuals. However, evidence on the BP-lowering effects of antihypertensive treatment in WCH is also limited and not entirely univocal. In a substudy of a trial on hypertensive patients aged 80 years or above [10] antihypertensive treatment showed an office and 24-h BP-lowering effect in patients with WCH [11]. In contrast, in an investigation based on about 2000 hypertensive patients of the European Lacidipine Study on Atherosclerosis (ELSA) [12], a 4-year antihypertensive treatment exhibited strikingly different effects on yearly office and ambulatory BP between WCH and sustained hypertension patients. That is, office BP was effectively reduced in both sustained hypertension and WCH throughout the treatment period whereas 24-h BP showed an effective and persistent reduction in sustained hypertension but no change or a slight progressive increase in the WCH group [13].

In the present study, we have analyzed the effect of antihypertensive drug treatment of sustained hypertension and WCH dyslipidemic patients in the Plaque Hypertension Lipid-Lowering Italian Study (PHYLLIS), that is, the only other trial in which, like in ELSA, office and ambulatory BP were measured over several years of BP-lowering drug administration [14]. We thought that this would allow us to determine whether the peculiar BP effects of treatment in WCH could be replicated in patients with different characteristics from those of the ELSA trial. In particular, as treatment was different in PHYLLIS and ELSA (calcium channel blocker or beta-blocker versus thiazide diuretic or ACE-inhibitor, respectively), we thought that this analysis would allow to determine whether the selective effect of treatment on office and out-of-office BP is independent on the treatment employed, and thus specific of the WCH condition itself.

## METHODS

### Study design and patients

The design and methods of the PHYLLIS study have been described in detail elsewhere [14,15]. Briefly, PHYLLIS was a multicenter (13 Italian hospitals), prospective, randomized, double-blind trial comparing the effect of antihypertensive and lipid-lowering drug treatment in untreated hypercholesterolemic hypertensive men and postmenopausal women (age 40–70 years) with asymptomatic carotid atherosclerosis and no previous cardiovascular events. Following an initial screening and after informed consent was obtained, patients underwent a 6-week washout under triple placebo during which they were maintained under the American Heart Association lipid-lowering diet. If, at the end of the wash-out period, office SBP and DBP were between 150 and 210 or between 95 and 115 mmHg, respectively; serum low-density lipoprotein (LDL) cholesterol was between 4.14 and 5.17 mml/l (160 –200 mg/dl) and serum triglycerides were less than 3.39 mml/l (<300 mg/dl), patients were randomized to (a) hydrochlorothiazide (HCTZ), 25 mg once daily with fosinopril (F) placebo and pravastatin (P) placebo; (b) F 20 mg once daily plus HCTZ placebo and P placebo; (c) HCTZ 25 mg and P 40 mg once daily plus F placebo and (d) F 20 mg and P 40 mg once daily plus HCTZ placebo. If, after 3 months, DBP was not reduced to less than 90 mmHg or by greater than 10 mmHg, open label Nifedipine Gastrointestinal Therapeutic System (GITS) 30 mg once daily was added, its dose being increased to 60 mg after an additional 3 or 6 months period in case of a persistent insufficiency of the BP response (see flow chart, Supplementary Fig. 1). Drugs were taken in the morning and the low-lipid diet used during the washout period was maintained throughout the study. Both patients and study personnel were blinded to the treatment measures. The primary outcome of the study was the rate of change in mean maximum intima–media thickness of the eight far and near walls of both distal common carotid arteries and bifurcations, which required carotid scans at randomization and, subsequently, at yearly intervals over the treatment duration of the study, which was on average 2.6 years. Secondary outcomes were changes in office BP, ambulatory BP, total serum cholesterol, LDL cholesterol, HDL cholesterol and triglycerides, with other biochemical values based on measurements performed every 6 months. The protocol was approved by the Ethic Committees of the Institutions involved.

### Blood pressure measurements

In each patient, trained personnel measured BP in the clinic before randomization to treatment, every 3 months after treatment initiation and then at the end of the trial. On each occasion, BP measurements were made with a mercury sphygmomanometer after the patient had been seated comfortably for at least 5 min. Heart rate was measured by the palpatory method after completion of the BP measurements. A 24-h ambulatory BP recording was obtained immediately before randomization, at yearly intervals during treatment and at the end of the trial, in each occasion within a week from office BP measurements. Office BP was measured in the morning and measurements were followed by assumption of the study drugs and the beginning of 24-h BP monitoring, which was preceded by a check that insured that ambulatory BP readings did not differ by more than 5 mmHg from the simultaneously obtained office readings. The participating centers had to use a validated ambulatory BP monitoring device (Spacelab 90207, Spacelab Inc, Redmond, Washington, USA) [16–18]. The devices were programmed to provide automatic readings every 15 min during the day (0600 h to midnight) and every 20 min during the night (midnight to 0600 h). Patients were instructed to attend at their usual activities during the recording period but to avoid strenuous exercise and to keep the arm extended and immobile during the cuff inflations. Ambulatory BP recordings were sent from each participating center to the coordinating center (Istituto Auxologico Italiano, Milan, Italy) to be checked for quality and analyzed centrally. Data were processed only if valid ambulatory BP readings were at least 70% of the expected numbers of reading and data from at least 21 h were available. As shown in Supplementary Table 1, the number of valid SBP readings was close to the number of expected SBP readings during the 24-h, the day and the night, this being the case for the baseline as well as for all yearly on-treatment recordings. Similar results were obtained for DBP (data not shown). Personnel involved in the BP analysis was kept blind of treatment assignment throughout the study.

### Data analysis

The three office BP measurements obtained at each visit were averaged, separately for SBP and DBP values. Averages were obtained also of 24-h SBP and DBP, and calculations were extended to office and 24-h heart rate values. On the basis of the baseline data obtained before randomization to treatment, patients were divided into a sustained hypertension group, that is, a group exhibiting an office SBP equal to or greater than 140 mmHg or an office DBP equal to or greater than 90 mmHg together with a 24-h SBP equal to or greater than 130 mmHg or 24-h DBP equal to or greater than 80 mmHg, namely the 24-h upper normality values indicated by guidelines [19]. And a WCH group, that is, a group exibiting an elevated office BP and a normal (<130/80 mmHg) 24-h BP. Calculations were extended to daytime and night-time mean BP and daytime and night-time BP variability (standard deviation and coefficient of variation of the daytime and night-time mean SBP or DBP). In the primary analysis, patients under F or HCTZ were pooled, while in additional analyses, they were considered separately as well as without or with the addition of P. Given its within-subject nature, comparisons between on-treatment and baseline BP values were made by the paired *t* test. Comparisons between the BP effects of treatment in sustained hypertension and WCH were made by analysis of variance with adjustment for the demographic (age and sex) and clinical baseline variables shown in Table 1 (ANCOVA), limited to those that were significantly different between the two groups with the exclusion of baseline BP values. A two-sided *P* less than 0.05 was taken as the level of statistical significance. Data analysis was carried out by a SAS software (SAS Institute, Cary, North Carolina, USA).

**TABLE 1:** Baseline demographic and clinical characteristics of sustained hypertension and white-coat hypertension patients of the Plaque HYpertension Lipid-Lowering Italian Study

Variables	SH	WCH	*P* value
*N*	361 (75.8%)	115 (24.2%)	–
Men (%)	42.7	33	0.0672
Age (years)	58.2 ± 6.8	58.2 ± 6.3	0.9425
Weight (kg)	70.3 ± 11.1	68.5 ± 11.6	0.1412
Smoking (%)	19.8	7.8	0.0028
Office SBP (mmHg)	160.7 ± 8.9	157.3 ± 8.5	0.0003
Office DBP (mmHg)	98.8 ± 4.2	96.8 ± 3.8	<0.0001
24-h SBP (mmHg	141.5 ± 11.8	119.9 ± 6	<0.0001
24-h DBP (mmHg)	87.5 ± 8.6	73.2 ± 5.1	<0.0001
Daytime SBP (mmHg)	144.7 ± 12.1	122.4 ± 6.4	<0.0001
Daytime DBP (mmHg)	90.3 ± 8.9	75.4 ± 5.6	<0.0001
Night-time SBP (mmHg)	129.4 ± 13.8	110.8 ± 8.5	<0.0001
Night-time DBP (mmHg)	76.6 ± 9.5	65.2 ± 6.3	<0.0001
Office heart rate (beats/min)	72.8 ± 7.6	72.7 ± 7.2	0.8881
24-h heart rate (beats/min)	73.7 ± 7.9	70.7 ± 7.9	0.0005
Total cholesterol (mg/dl)	261.6 ± 25.4	264.4 ± 27.4	0.3209
LDL cholesterol (mg/dl)	180.5 ± 19.6	183 ± 20.5	0.2394
HDL cholesterol (mg/dl)	52.7 ± 13	53.6 ± 14.7	0.5549
Triglycerides (mg/dl)	141.5 ± 56.5	141.1 ± 58.4	0.9417
Serum creatinine (mg/dl)	0.9 ± 0.2	0.9 ± 0.1	0.0746
Blood glucose (mg/dl)	94.5 ± 13	91.7 ± 12.4	0.0474
Diabetes mellitus (%)	1.8	0	0.3432

The symbol ± refers to the standard deviation of the mean. HDL, high-density lipoprotein; LDL, low-density lipoprotein; SH, sustained hypertension; WCH, white-coat hypertension.

## RESULTS

### Baseline data

Four hundred and seventy patients met the criteria for data analysis, of whom 75.8% and 24.2% were defined as sustained hypertension and WCH patients, respectively. Table 1 shows that gender representation, age, weight, lipid profile, serum creatinine, blood glucose, prevalence of diabetes mellitus and office heart rate values were not significantly different in the two groups. In both groups, office BP values were elevated (SBP ≥140 mmHg or DBP ≥90 mmHg) the elevation being slightly but not significantly greater in the sustained hypertension than in the WCH group. The 24-h BP was elevated (SBP ≥130 mmHg or DBP ≥80 mmHg) in the sustained hypertension but not in the WCH group, which also showed a modestly lower 24-h heart rate value compared with the sustained hypertension group.

### Blood pressure effects of antihypertensive treatment

**FIGURE 1:**
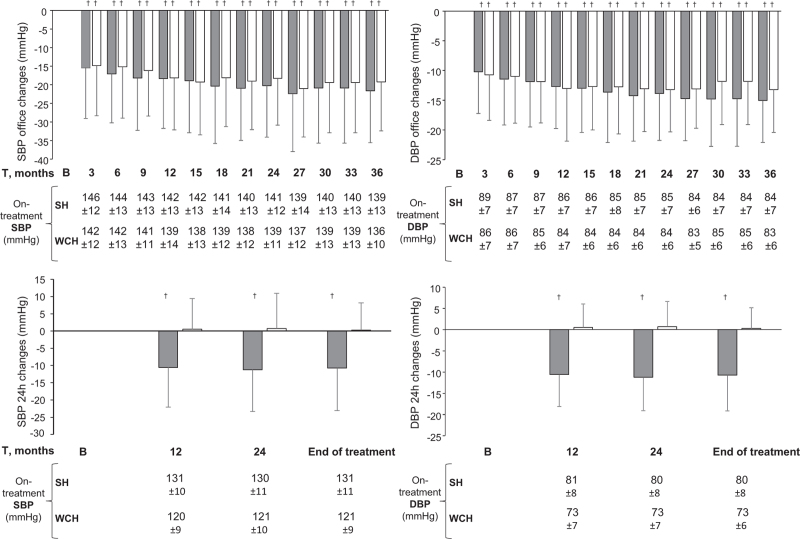
Reduction (mean ± standard deviation) of office and 24-h (h) SBP and DBP from baseline during treatment with fosinopril or hydrochlorothiazide with or without pravastatin (data pooled) in sustained hypertension (grey histograms) and white-coat hypertension (white histograms). Data are shown as means ± standard deviation for different treatment times after randomization to one or the other drug. † refers to statistical significance (*P* < 0.0001) of BP reduction in the different treatment periods compared with baseline; absolute on-treatment SBP values are also shown. SH, sustained hypertension; WCH, white-coat hypertension.

**FIGURE 2:**
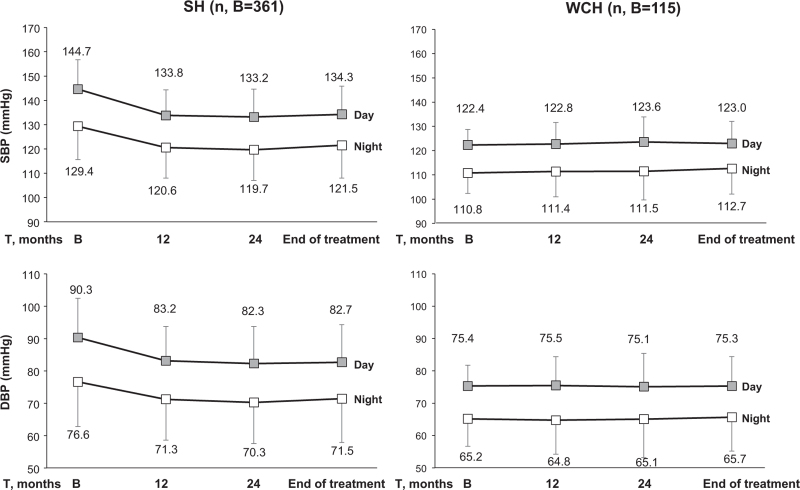
Day and night mean (± standard deviation) SBP and DBP values at baseline (B) and after 12 months, 24 months and end of treatment (T) with fosinopril or hydrochlorothiazide with or without pravastatin (data pooled). Symbols and abbreviations as in Fig. 1.

**FIGURE 3:**
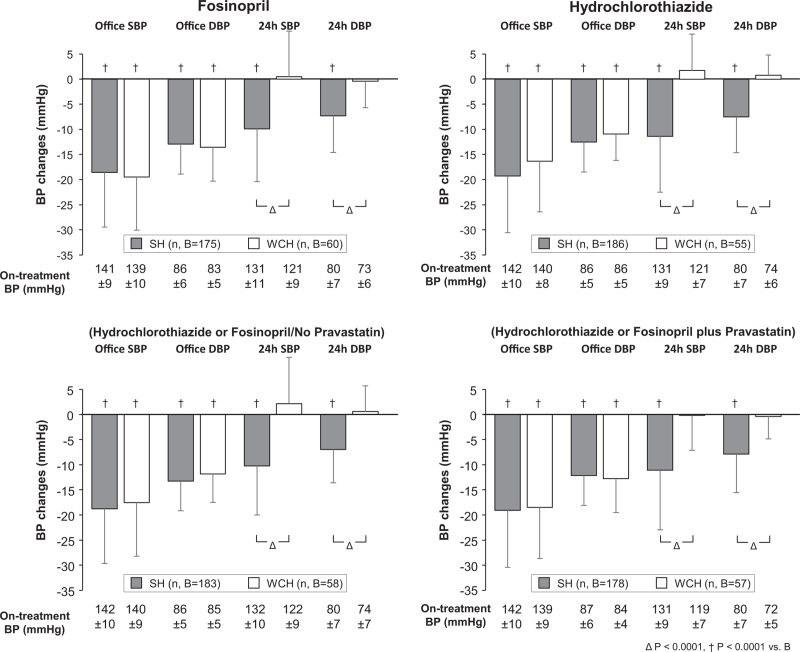
Reduction (mean± standard deviation) of office and 24 h SBP and DBP from baseline in patients with sustained hypertension and white-coat hypertension (1) separately treated with fosinopril or hydrochlorothiazide, regardless the concomitance of pravastatin treatment (top panels) and (2) separately treated with fosinopril or hydrochlorothiazide (data pooled) in absence or with the addition of pravastatin (bottom panels). Data obtained after 1 year, 2 years and at the end of treatment were averaged. Mean (± standard deviation) absolute on-treatment SBP and DBP values are reported at the bottom. Symbols and abbreviations as in Figs. 1 and 2.

As reported in the original PHYLLIS paper [15], antihypertensive monotherapy was employed in the vast majority of the patients, the addition of nifedipine GITS being required in only 20.2, 19.8, 15.8, and 15.8% of the a, b, c and d groups, respectively. As shown in Fig. 1, antihypertensive treatment was associated with an early marked reduction of either SBP and DBP, which persisted virtually unchanged throughout the treatment period. This was the case also in the sustained hypertension group and the magnitude of the office SBP and DBP treatment-induced changes was only slightly and usually not significant different between the sustained hypertension and WCH groups also after adjustment for the demographic and clinical variables (Fig. S2) shown in Table 1. This was not the case; however, for 24-h SBP and SBP, which fell persistently from baseline in sustained hypertension while showing no change in WCH (Fig. 1), the pattern being identical also after adjustment for the variables of Table 1 (Fig. S2). In sustained hypertension, but not in WCH, antihypertensive treatment consistently reduced either the higher daytime and the lower night-time on-treatment SBP and DBP values (Fig. 2). The results were similar when data analysis considered separately patients randomized to F or HCTZ (Fig. 3) or patients randomized to F and HCTZ treatment pooled without or with the addition of P (Fig. 3).

**FIGURE 4:**
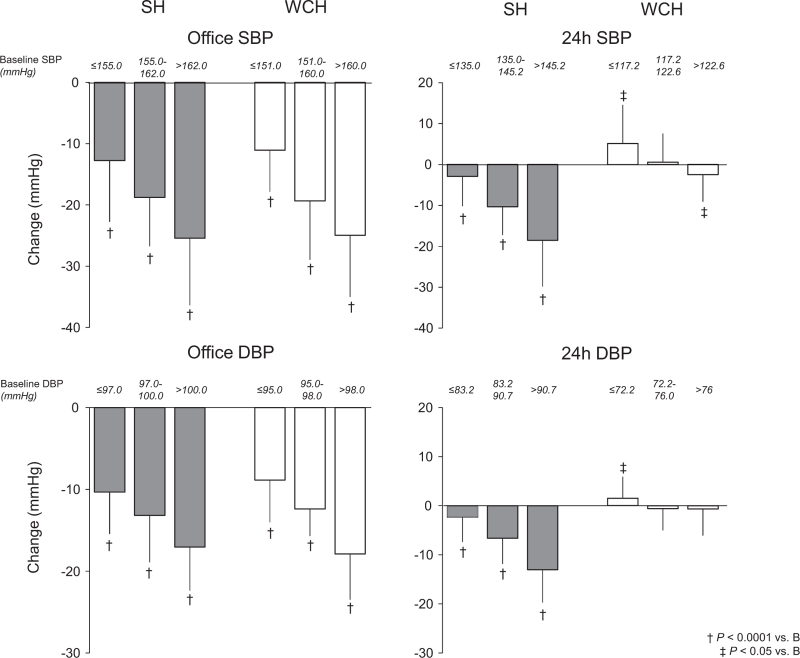
Relationship between baseline office or 24-h SBP or DBP and the respective SBP or DBP reduction induced by antihypertensive treatment in the sustained hypertension and white-coat hypertension groups. Data are shown for progressively greater tertiles of SBP and DBP baseline values. Histograms refer to the four treated groups pooled. SBP or DBP reductions (± standard deviation) were calculated as means of the reductions seen after 1 year, 2 years and at the end of treatment. Symbols below the histograms refer to the statistical significance of the SBP or DBP reductions from baseline. Baseline BP values are also shown. Other symbols and abbreviations as in previous figures.

**FIGURE 5:**
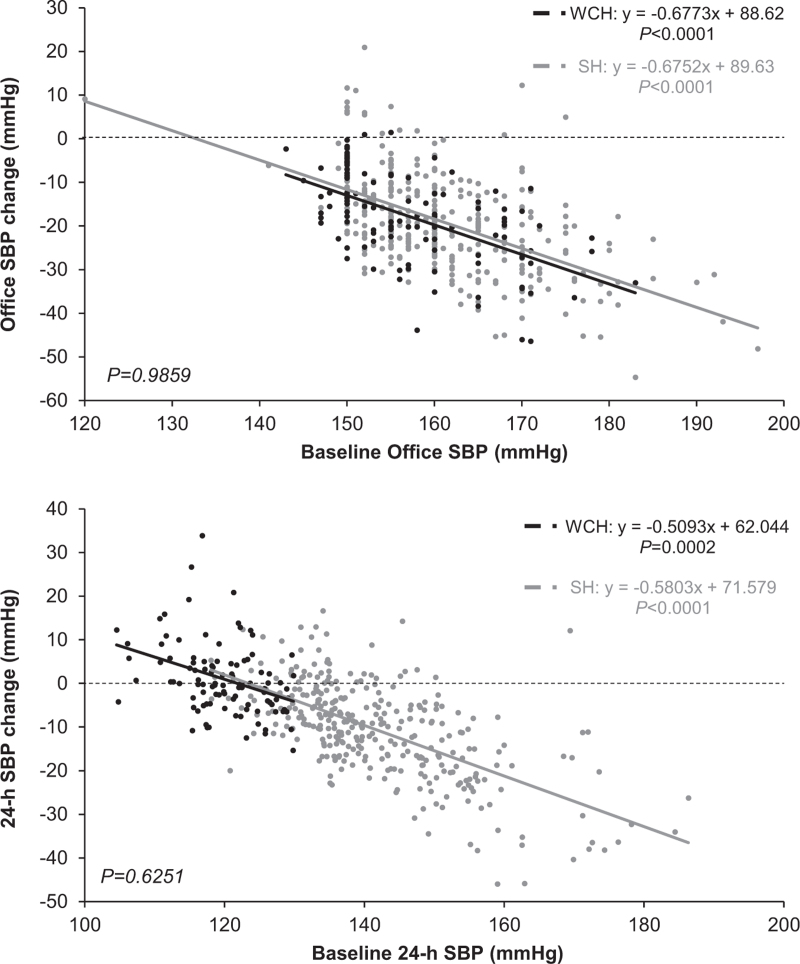
Relationship between baseline office or 24-h SBP and the respective SBP reduction with treatment in the sustained hypertension and white-coat hypertension groups shown for individual patients. In each patient, data represent the average SBP reduction after 1 year, 2 years and at the end of treatment. Patients under F or HCTZ with or without P were pooled. Other abbreviations and symbols as in previous figures.

Figure 4 shows the relationship between baseline office or 24-h BP and the corresponding BP reductions with antihypertensive treatment (F and HCTZ data pooled).. In both the sustained hypertension and the WCH group, the treatment-induced reduction of office SBP or DBP increased steeply from the tertile with the lowest to the tertile with the highest office baseline BP value. In sustained hypertension, this was the case also for the treatment-induced reduction of 24-h SBP or DBP whereas in the WCH group, the 24-h SBP or DBP reduction was almost undetectable at any 24-h baseline SBP or DBP tertile. However, even in this case, there were some between tertile differences, that is, a very small but significant 24-h BP reduction at the highest baseline 24-h BP tertile and a very small but significant 24-h BP increase at the lowest 24-h BP tertile. The tendency of 24-h BP to increase at the lowest baseline 24-h tertile is even more visible when the data are shown for all individual patients (Fig. 5).

### Twenty-four hour blood pressure variability

**FIGURE 6:**
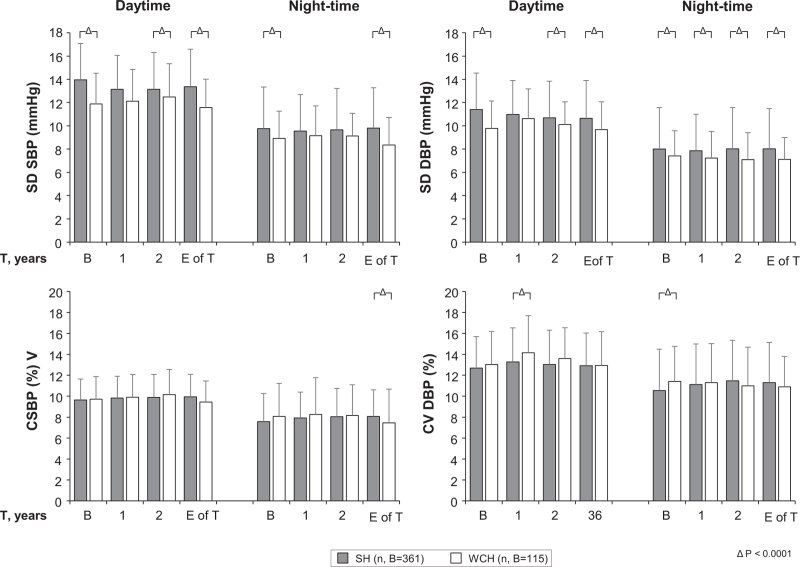
Standard deviation and coefficient of variation of mean day and night-time SBP and DBP values at baseline and after 1 year, 2 years and at the end of treatment in the sustained hypertension and white-coat hypertension groups. Data were pooled for the four treated groups and are shown as means ± standard deviation. E of T, end of treatment. Symbols and abbreviations and explanations as in previous figures.

Figure 6 shows that both at baseline and during treatment, the day and the night SBP standard deviation was often modestly but significantly greater in the sustained hypertension than in the WCH group. Between-group differences were always negligible when BP variability was expressed as coefficient of variation, which in both sustained hypertension and WCH was never modified by treatment.

## DISCUSSION

Our analysis of the PHYLLIS study provides three main results. First, in WCH patients, antihypertensive treatment caused an early, marked and sustained reduction of office SBP and DBP that was substantially similar to that seen in patients with sustained hypertension. Second, while in sustained hypertension, the treatment-induced office SBP and DBP reduction was associated with a clearcut sustained reduction of 24-h, daytime and night-time SBP and DBP, in WCH, no ambulatory SBP and DBP reduction occurred. Third, the effects seen on office and 24-h BP were similar in patients treated with F or HCTZ, with or without the concomitance of lipid-lowering treatment. This confirms the results obtained in the ELSA study [13], which also showed antihypertensive treatment to effectively and durably reduce both office and ambulatory BP in sustained hypertension while having an effective and durable antihypertensive influence on office BP only in WCH. Because in the ELSA study treatment consisted of atenolol or lacidipine while in the PHYLLIS study the administered drugs were a thiazide, an ACE-inhibitor and a statin it also adds to previous data that the peculiar BP effects of antihypertensive treatment in WCH (large office but no ambulatory BP reduction) extend to virtually all major antihypertensive agents, and that thus, they do not depend on the type or mechanism of the treatment (or co-treatment) employed but reflect the response of the WCH phenotype to any BP-lowering intervention.

Other results of our study deserve mentioning. First, our study shows that in both WCH and sustained hypertension, the treatment-induced reduction of office SBP and DBP was closely associated with baseline BP levels, that is, in both conditions the higher was the elevation of baseline office BP the greater was its subsequent treatment-induced reduction. They further show that a similar association characterized the 24-h SBP or DBP reduction and the corresponding baseline 24-h BP values although at a glance this was limited to the sustained hypertension group. A closer look, however, reveals that WCH was qualitatively not extraneous to this phenomenon as within the normal baseline BP values that characterize this condition, patients in the higher tertile (SBP 123 mmHg) exhibited a small but significant BP reduction whereas patients in the intermediate tertile showed no BP change and patients in the lowest tertile (SBP 117 mmHg) exhibited a small but significant BP rise. These observations, also in line with those obtained in the ELSA study [13], allow to conclude that treatment-induced BP reductions and baseline BP values are closely associated regardless whether BP values are obtained by spot measurements in the office or over the 24-h. This is the case for the entire baseline BP range, including the portion defined as 24-h BP normality that characterizes WCH. In practical terms, this means that in WCH people antihypertensive treatment may be expected to cause some 24-h BP reduction if baseline 24-h BP is close to the upper limit of normality (130/80 mmHg) but to have no effect or even be associated with a BP increase when baseline values are in the lowest portion of the normality range. Third, we do not have an explanation for the observation that at lowest baseline 24-h BP, antihypertensive treatment may be accompanied by a BP increase, except for the possibility that this reflects a regression to the mean. It is interesting to note, however, that an explanation based on regression to the mean implies that regression to the mean involved values that were already a mean of a considerable number of individual values. Fourth, both at baseline and during treatment, WCH patients showed BP standard deviations that were smaller than those seen in sustained hypertension, especially during the day, indicating that short-term BP oscillations may be less pronounced in WCH than in sustained hypertension. This is likely to be accounted for by the lower ambulatory BP that characterizes WCH as 24-h BP standard deviation is proportional to mean BP values [20] and in the present study, the standard deviation differences between sustained hypertension and WCH disappeared when variability was expressed as variation coefficient, a procedure that eliminates the variability component due to mean BP [20]. Regardless the mechanisms, a lower 24-h BP variability may contribute to the lower cardiovascular risk that has been repeatedly reported for WCH compared with sustained hypertension patients [21–24] as the magnitude of the BP variations that occur during the day and night 24-h have an independent adverse influence on the risk of cardiovascular outcomes [25]. Fifth, it is of interest that in the WCH participants of the PHYLLIS study addition of a calcium channel blocker to F or HCTZ was rare (15–20% of the study population), suggesting that in this condition, a marked office BP reduction can be frequently obtained with a single drug only, with no need to resort to more complex and side-effects-generating treatments [26]. This, may be a factor to consider if the doctor's decision is to give these patients an antihypertensive drug treatment.

The results of our study are relevant to the question whether the increased cardiovascular risk that characterizes WCH patients compared with normotensive individuals deserves use of antihypertensive drugs. Most hypertension guidelines recommend to limit routine intervention to a closer follow-up and lifestyle changes and to only consider administration of BP-lowering agents if cardiovascular risk is high or there is organ damage [19,27]. However, there are no therapeutic studies in which the effect of antihypertensive treatment of WCH patients on cardiovascular outcomes was appropriately compared with the effect of treatment in sustained hypertension. We can speculate that in this context, the possibility of a protective effect of antihypertensive treatment should not a priori be excluded because, as shown in the present study, in WCH antihypertensive treatment can lower an ambulatory BP close to the upper normality limit. Furthermore, previous studies have shown that an increase of office BP may have detrimental effect independently on ambulatory BP values. For example, in WCH office BP predicted the increased risk of new onset sustained hypertension after adjustment for 24-h BP [19]. Furthermore, an increase of the office BP value increased the risk of cardiovascular outcomes for the same 24-h BP level [28].

Our study has strengths and limitations. One strength is the high quality of the ambulatory BP recordings as well as the multiple collection of office and ambulatory BP values over years. Another is that in the present study, confirmation of the results of the ELSA study was obtained with different antihypertensive drugs. The limitations are that the number of patients with WCH was not large. Furthermore, as in previous studies, the number of cardiovascular outcomes provided by the PHYLLIS study was small, which made it impossible to directly analyze the relationship between the peculiar effects of treatment on the BP values of WCH individuals and the risk of cardiovascular outcomes. In other words, whether a treatment-induced reduction of office BP that only marginally involves out-of-office BP can lead to cardiovascular protection. Finally, the PHYLLIS trial was randomized for the type of treatment employed and not for treatment of WCH versus sustained hypertension. Thus, the results have an observational nature that does not allow confounding to be completely excluded. On the other hand, the availability of serial on-treatment ambulatory and office BP measurements (rather than measurements at a single occasion only [9,11]) makes the conclusion that in WCH, antihypertensive treatment lowers office but not out-of-office BP robust. Together with the results of the ELSA study, the present findings also allow to conclude that in WCH, the peculiar BP-lowering pattern that accompanies antihypertensive treatment is independent from the type of treatment employed.

## ACKNOWLEDGEMENTS

### Conflicts of interest

There are no conflicts of interest.

## Supplementary Material

Supplemental Digital Content
