# Supplementary material for: Antihypertensive drug treatment in white-coat hypertension: data from the Plaque HYpertension Lipid-Lowering Italian Study
Source: J Hypertens. 2022 Jul 25;40(10):1909–17. doi: 10.1097/HJH.0000000000003176 (PMC10860885; doi:10.1097/HJH.0000000000003176)
Supplement: Supplemental Digital Content [file jhype-40-1909-s001.docx]

**Figure S1 -** Design of the Plaque Hypertension Lipid-Lowering Italian Study (PHYLLIS) trial. HCTZ:hydrochlorothiazide. QD:once a day. Drugs or placebo were given once daily. S:supplementary

**
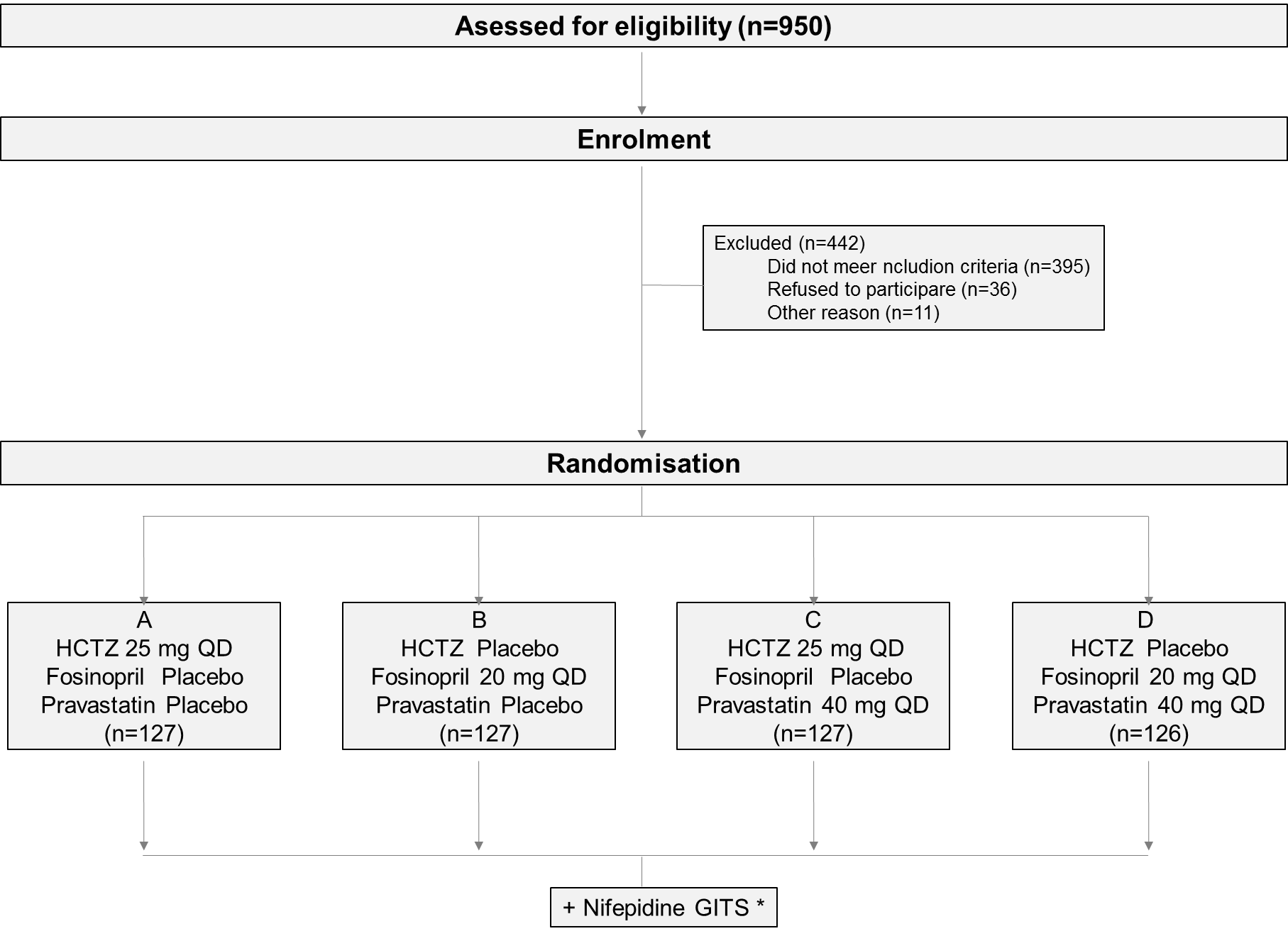
**

* open-label nifedipine gastrointestinal therapeutic system (GITS), 30 mg once daily, was added after 3 months to be eventually increased to 60 mg after 6 months

**Figure S2** Reduction (means ± standard error) of office and 24h SBP and DBP from baseline in SH and WCH during treatment with F or HCTZ with or without P. Data refer to the same patients of Figure 1 after adjustment of BP values for demographic and baseline clinical differences between SH and WCH reported in Table 1.The symbol ∆ refers to the adjusted P(P< 0.0001)values by ANOVA. No statistical symbol means no statistical significance Symbols and abbreviations as in Figure 1.

**
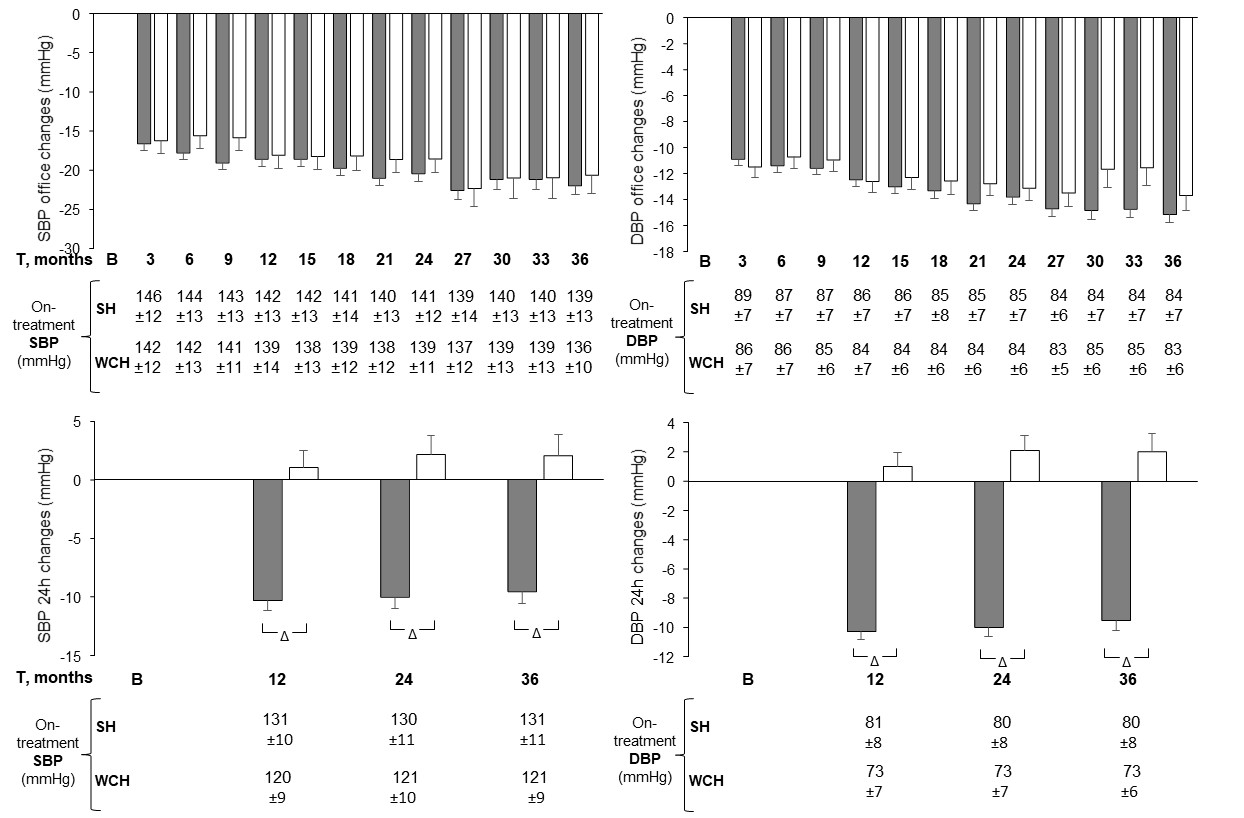
**

**Table S1**. Number of valid BP readings over the 24 hours, the daytime and the nighttime in all patients and in white-Coat hypertension (WCH) and sustained hypertension (SH). Based on the intermittency of the automatic BP readings and the duration of the day and night periods (18 and 6 hours) the expected number of BP values for each ambulatory BP monitoring were 90 for the 24 hours, 72 for the daytime and 18 for the nighttime.

|  |  | **Number of measurements** | | |
| --- | --- | --- | --- | --- |
|  |  | **24h** | **Daytime** | **Nighttime** |
| **Patients** | **Visit** |  |  |  |
| **All Patients** | Baseline | 78.9±9.5 | 62.3±7.9 | 16.6±3 |
|  | Treatment (Years) |  |  |  |
|  | 1 | 78.7±9.1 | 62.1±7.6 | 16.6±2.9 |
|  | 2 | 79.8±8.8 | 62.3±8.0 | 17.5±2.2 |
|  | 3 | 79.4±8.7 | 61.8±7.7 | 17.6±2.2 |
| **BP phenotype** | |  |  |  |
| WCH (N=115) | Baseline | 76.0±9.8 | 59.7±8.4 | 16.3±3 |
|  | Treatment (Years) |  |  |  |
|  | 1 | 75.7±11.8 | 59.5±9.7 | 16.2±3.3 |
|  | 2 | 78.2±10.0 | 60.4±8.9 | 17.8±2.7 |
|  | 3 | 77.6±8.9 | 60.0±7.9 | 17.6±2.1 |
|  |  |  |  |  |
| SH (N=361) | Baseline | 79.8±9.2 | 63.1±7.5 | 16.7±3.0 |
|  | Treatment (Years) |  |  |  |
|  | 1 | 79.6±8.0 | 62.9±6.6 | 16.7±2.7 |
|  | 2 | 80.2±8.3 | 62.8±7.6 | 17.4±2.0 |
|  | 3 | 79.8±8.6 | 62.3±7.6 | 17.5±2.2 |

Data are shown as means±standard deviations. BP: blood pressure, SBP:systolic blood pressure
